# Estimating the evidential value of significant results in psychological science

**DOI:** 10.1371/journal.pone.0182651

**Published:** 2017-08-18

**Authors:** Balazs Aczel, Bence Palfi, Barnabas Szaszi

**Affiliations:** 1 Institute of Psychology, ELTE, Eotvos Lorand University, Budapest, Hungary; 2 School of Psychology, University of Sussex, Brighton, United Kingdom; 3 Sackler Centre for Consciousness Science, University of Sussex, Brighton, United Kingdom; 4 Doctoral School of Psychology, ELTE, Eotvos Lorand University, Budapest, Hungary; Tilburg University, NETHERLANDS

## Abstract

Quantifying evidence is an inherent aim of empirical science, yet the customary statistical methods in psychology do not communicate the degree to which the collected data serve as evidence for the tested hypothesis. In order to estimate the distribution of the strength of evidence that individual significant results offer in psychology, we calculated Bayes factors (BF) for 287,424 findings of 35,515 articles published in 293 psychological journals between 1985 and 2016. Overall, 55% of all analyzed results were found to provide BF > 10 (often labeled as strong evidence) for the alternative hypothesis, while more than half of the remaining results do not pass the level of BF = 3 (labeled as anecdotal evidence). The results estimate that at least 82% of all published psychological articles contain one or more significant results that do not provide BF > 10 for the hypothesis. We conclude that due to the threshold of acceptance having been set too low for psychological findings, a substantial proportion of the published results have weak evidential support.

## Introduction

The reliability of evidence published in psychological science recently became a major concern as a high rate of published experiments failed to generate significant results upon replication [*1*], including some classic textbook studies [*2*]. While most often this low replicability of statistically significant findings is attributed to publication bias [*3*], questionable research practices [*4*] and flawed statistical analyses [*5*], replicability is also conditional on the degree to which significant results in themselves constitute evidence for an experimental hypothesis. To explore this question, we provide a general overview of the evidential value of significant results in psychological journals.

In general, empirical scientists form hypotheses about the world and collect data to evaluate these hypotheses. In order to quantify their evidence, researchers in psychology and other social sciences use their data to distinguish true effects from random chance. In practice, they overwhelmingly use null-hypothesis significance testing (NHST), which estimates the probability of receiving an observation (or more extreme ones) if the null-hypothesis of no effect is true. If this probability, the p-value, is equal to or less than a preset threshold (customarily 5%) then researchers reject the null-hypothesis and claim support for the effect. Since the development of this procedure in the early 20^th^ century [*6*], social sciences have been relying on this logic when providing evidence for their theoretical hypotheses.

Nevertheless, it is a common misunderstanding that the NHST method can quantify the evidence for a hypothesis [*7*]. For example, p = .05 is often misinterpreted as 5% probability that the null-hypothesis is true or that chance alone produced the effect [*8*]. Unfortunately, however, the NHST approach cannot be used to directly evaluate the hypothesis since it estimates the probability of statistical significance under the assumptions of the null-hypothesis, but ignores the probability of statistical significance under the alternative hypothesis. In fact, the long-term rate of false discovery depends not just on the p ≤ 0.05 rule, but also on the prevalence of the effects and the power of the tests. Taking these into account, the false discovery rate of NHST tests is estimated to be much higher than 5% [*9*]. In sum, although p < .05 results have been interpreted as convincing evidence in support of psychological effects, statistical logic does not admit such use of the method.

One way to quantify evidence is to assess how much the data support one or a competing hypothesis. The Bayes factor is a tool that can be used to calculate the ratio of the likelihood of the hypotheses in light of the data, thus expressing how much the observation should change our beliefs about the hypotheses. Recent developments in the method [*10*–*12*] allow researchers to effectively employ Bayes factor analysis techniques to quantify the strength of the evidence against, as well as for, the null-hypothesis. A major difference from the traditional statistics is that this Bayes factor computation requires specifying what the hypotheses predict, known as the priors. While the choice of prior can be debated and gives the impression of subjectivity, there are two main reasons why this feature of the method should not be a major concern. First, it is important to see that the need to specify the predictions of the theory is not a limitation, but a basic requirement of science [*13–14*]. Second, applications of the method, such as this work, show that the results are often robust to the choice of reasonable priors [*15*].

While the Bayes factor is a continuous scale between 0 and positive infinite, customary labels are often used to categorize the strength of evidence [*16*](*Table 1*). Assuming the reader has no prior expectations regarding the outcome of the research, by Bayes factor of 1 we can infer that the evidence is equally in favor of the null and alternative hypotheses, a clearly insensitive case. The range of Bayes factors between 1 and 3 has been suggested as a region for anecdotal evidence for the alternative hypothesis, while Bayes factors between 1 and 1/3 indicate anecdotal evidence towards the null [*17*]. Bayes Factor 3 indicates that the odds are 3:1 for the alternative hypothesis, allowing 75% confidence in the results. The next level is the *moderate evidence*, which is used for Bayes factors between 3 and 10, and Bayes factors between 1/3 and 1/10. To be able to claim strong support, Bayes factor > 10 and < 1/10 are considered to be the threshold for H1 and H0, respectively [*17*].

**Table 1:** Bayes factor evidence categories and the corresponding Bayes factor intervals [*16*–*17*].

Bayes factor evidence categories	Value of the Bayes factor
Strong support for H1	> 10
Moderate support for H1	3–10
Anecdotal support for H1	1–3
Equal support for the hypotheses	1
Anecdotal support for H0	1/3–1
Moderate support for H0	1/10–1/3
Strong support for H0	< 1/10

Different categories have been suggested as a requirement for a result to be taken as evidential. Originally, Jeffrey proposed that Bayes factor 3 mostly correspond with the level of evidence that is usually obtained with conventional significance testing [*16*]. Others suggested that the conventional threshold is too liberal and Bayes factor 6 is more efficient in correctly capturing the effect [*18*] and 10 provides more compelling evidence [*18–19*]. Nevertheless, it is important to emphasize that it would be a misuse to blindly apply Bayes factors as hard cut-off levels. Instead, these indices should inform the reader about the degree to which the observations support one compared to another hypothesis. Although researchers often set expected levels of evidence for their decisions, the relative nature of the support should never be disregarded.

While there is a functional correspondence between p-values and Bayes factors [*20*], the question remains as to how much evidence is provided by individual significant results in psychology. A previous investigation compared all p-values and the computed Bayes factors of 855 t-tests published in two experimental journals in 2007 [*21*]. Those results demonstrated that if p-values are misinterpreted as evidence then they mostly agree with the Bayes factor results about the direction of their support, yet they systematically overestimate the strength of the evidence against the null-hypothesis. It is a question, however, whether this sample of the two journals can be generalized to all domains of psychology. To extend this analysis and assess the overall picture about the strength of evidence in psychological science, we computed Bayes factors with additional robustness analyses for 287,424 significant t- and F-tests using a text-mined dataset of 35,515 psychological articles published between 1985 and 2016 [*22*]. This dataset contains all reported t- and F-tests from 293 psychological journals from this period of time, representing a substantial corpus of all published research of the field. Here, we use this sample to estimate the strength of evidence that individual significant results provide for the alternative hypothesis.

## Methods

The analysis plan of this study was preregistered on osf.io/5rf2x prior the conduct of all the reported analyses. For the analyses, we used a dataset collected by Hartgerink [*22*]. This dataset contains the metadata and statistical parameters of 688,112 null-hypothesis test results text-mined from 50,845 articles published in 321 psychology journals between 1985 and 2016.

For our main analysis, we limited our database to the *t*, *and F* statistics which are the most frequently used statistics in psychology [*22*] and we assumed an independent sample design, the most lenient condition for supporting the alternative hypothesis. First, we selected 403,929 test statistics (the 169,984 t-tests, the 233,945 F-tests where the value of the first degrees of freedom was one). Using the recalculated p-values we excluded the non-significant test statistics (109,470 results), as the question of our interest was to calculate the degree of evidence behind the significant results. To exclude ambiguous cases from our analyses, we filtered out those results which did not provide the exact values of the test statistics (374 results), as well as the one-sided tests (4878 results), having no information about the direction of the hypotheses of those tests. Furthermore, we removed those test statistics where the recalculated p-values changed whether or not the results were statistically significant using the 0.05 decision threshold (705 results). Next, we rounded up all of the non-integer degrees of freedoms of the t-tests as we assumed that these scores were reduced by a correctional process (e.g., Welch t-test). We calculated the square root of the F-values and analyzed them as t statistics thereafter. Afterward, we calculated the sample sizes of the results from the reported degrees of freedom assuming independent-sample design. Finally, we took out the results where the overall sample size did not exceed 5 to exclude the extreme cases (486 t-tests, 592 F-tests). After these selections, 114,272 t-tests and 173,152 F-tests remained in our dataset.

To compute the Bayes factors corresponding to the t- and F-tests, we employed the *ttest*.*tstat* function of the BayesFactor R package [*23*]. The calculation of the Bayes factors requires specification of the predictions (prior distribution) of the null- and the alternative models [*15*, *24*]. In cases where the effect size of the alternative model can be high, it is suggested to employ a two-tailed Cauchy distribution centered on zero as a prior distribution of the alternative hypothesis [*15*]. The scaling of this prior distribution can influence the calculated value of the Bayes factors, and so the level of evidence favoring the hypotheses. Accordingly, for smaller effects a high-scaled prior distribution can underestimate the degree of positive evidence compared to a low-scaled prior. To determine the scaling factor of the Cauchy distribution, we applied the median effect size (d = 0.93) of a collection of 100,000 significant statistical tests, extracted from 10,000 psychology articles, as a rough estimate [*25*]. As this calculated value could be influenced by a small number of extremely high effect sizes, we decided to employ the so called default *JZS prior* (√2/2) for our main analyses, which is more lenient towards smaller effect sizes [*26*]. This value is the default scaling factor of the *ttest*.*tstat* function and is considered a ‘medium’ scaling factor [*23*]. To explore the robustness of our results, we repeated the analyses with different sizes and shapes of the prior distribution (See S1 Supplementary methods and results). Using the resulting Bayes factor values, we categorized each test result into the corresponding Bayes factor evidence category of Table 1.

## Results

**Fig 1:**
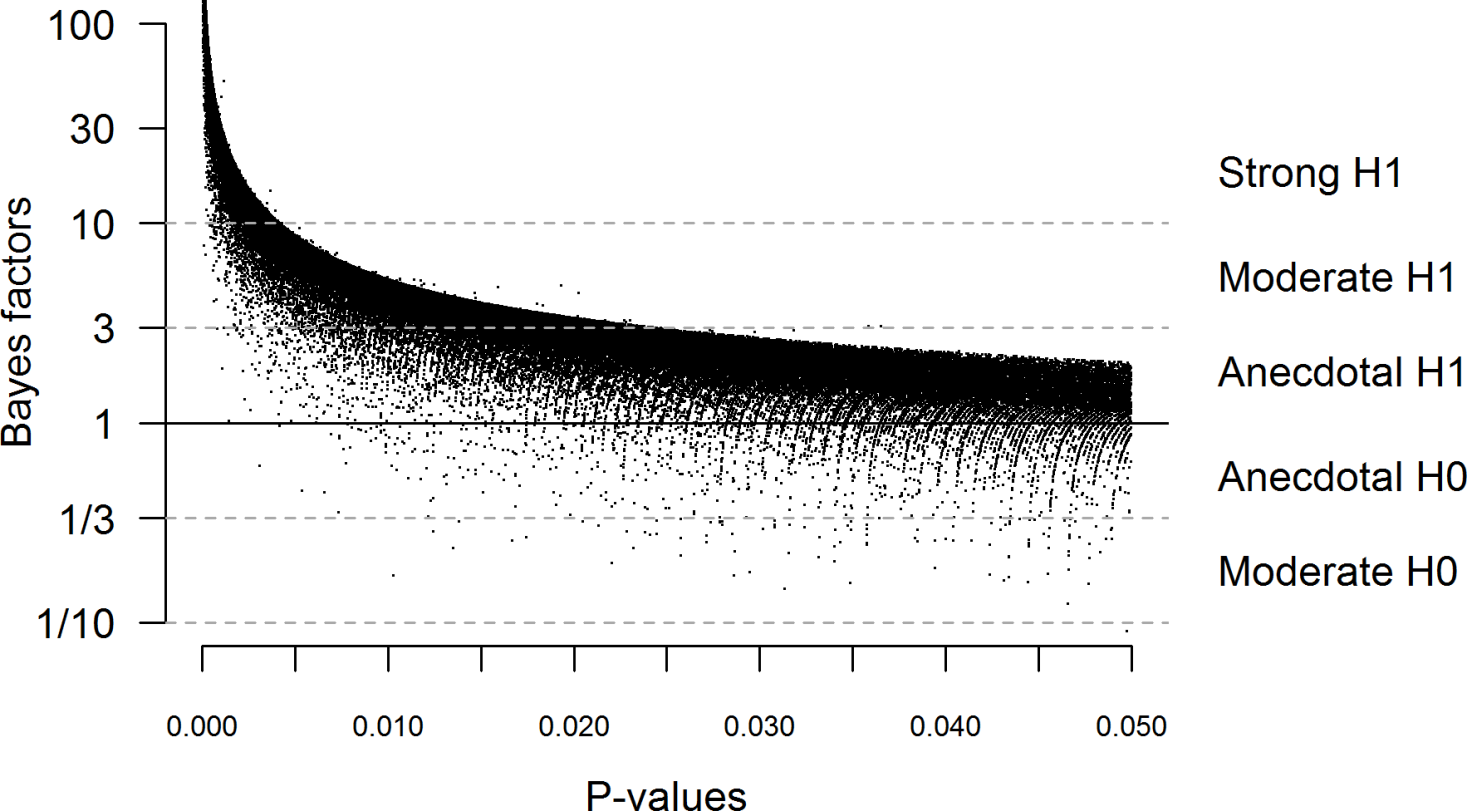
The relationship between published significant p-values and the corresponding Bayes factors for the t- and F- test results. The Bayes factors were calculated with medium scaled prior distribution assuming independent-samples design for t- and F-test results. The stripes on the plot are the results of the general custom of rounding the t- and F-values to two decimals.

**Fig 2:**
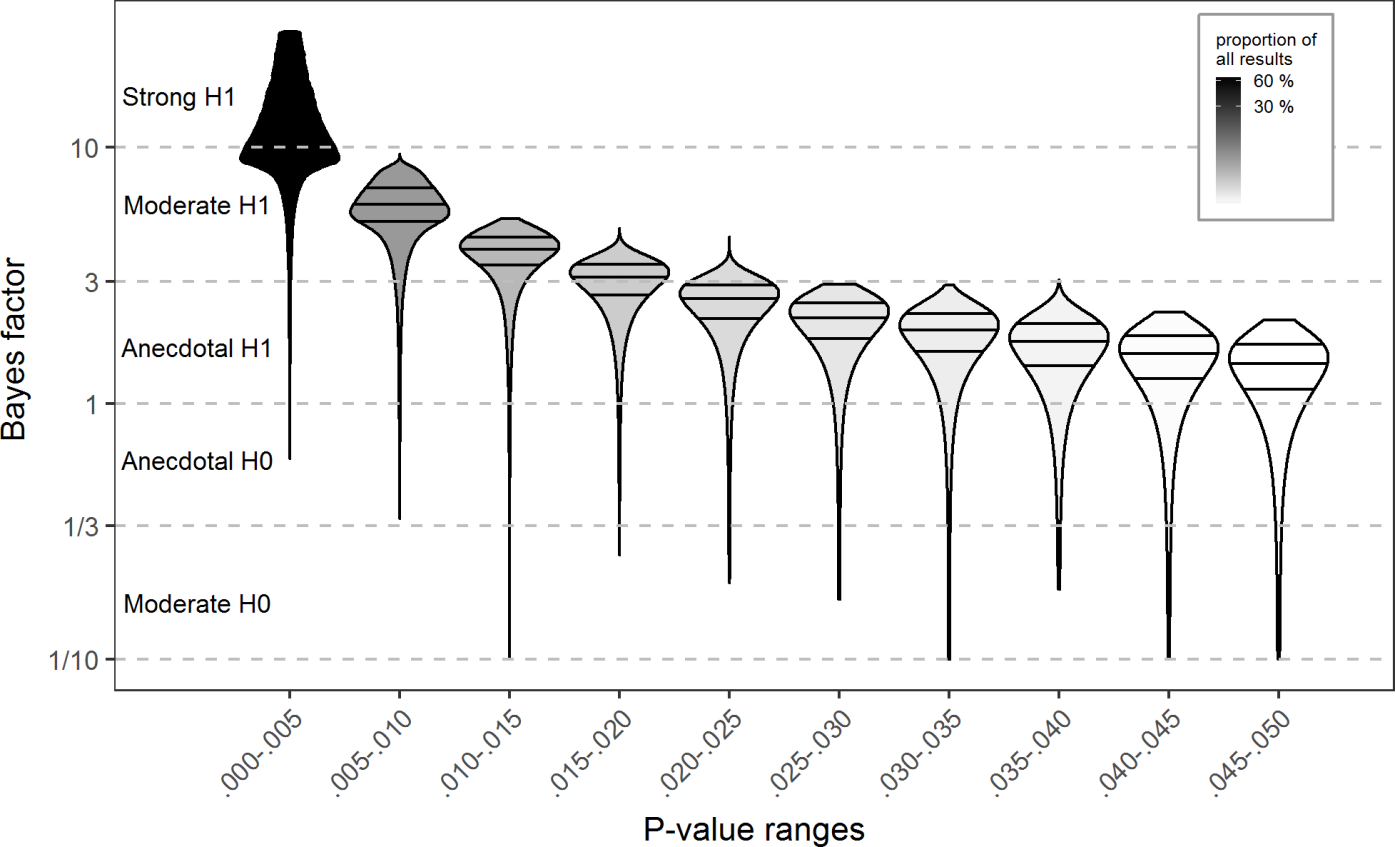
Density plots with quartile lines for the Bayes factors in ranges of published significant p-values. The corresponding Bayes factors were calculated on the t- and F- test results with medium scaled prior distribution assuming independent sample design. For visualization purposes, the leftmost plot depicts only the lowest quartile of the corresponding Bayes factors.

The results show that overall only 54.7% of all analyzed results provide strong evidence (B = 10) for the alternative hypothesis, while half of the remaining results do not pass the level of even anecdotal evidence (B = 3) (Table 2). As illustrated in Figs 1 and 2, due to the functional relationship between p-values and Bayes factors [*20*, *26*], there is an upper bound on the potential Bayes factors corresponding to p-values. As a result, findings with p > 0.005, cannot provide strong evidence for the alternative hypothesis. Of note, even among those results with p < 0.005, 6% still fail to reach the level of strong evidence. Results remain robust with different prior distribution settings and when assuming paired sample tests (see S1 Supplementary methods and results). Repeating the same analyses on the significant correlation findings in the collection (N = 15,026), the pattern of results differed only slightly, with 59.6% of results reaching the level of strong evidence, half of which remained in the anecdotal evidence region (S1 Supplementary methods and results). Considering just the t-, and F- statistics, the results indicate that at least 82% of all psychological articles contain at least one significant result which does not provide strong evidence. This result highlights that the rejection of the null-hypothesis based on significant p-values gives only ambiguous support for the alternative hypothesis.

**Table 2:** The proportion of significant t- and F-test results in the different Bayes factor evidence categories. The corresponding Bayes factors were calculated with medium scaled prior distribution assuming independent-samples design.

	Strength of evidence						
	Strong H1	Moderate H1	Anecdotal H1	Anecdotal H0	Moderate H0	Strong H0	Total
N/row total (%)	**54.7%**	**21.4%**	**22.9%**	**1.0%**	**0.0%**	**0.0%**	**100.0%**
N	157,184	61,579	65,677	2,861	116	7	287,424

## Discussion

As Figs 1 and 2 depict, the strength of evidence that significant results provide vary greatly. While there is an upper bound to the Bayes factors corresponding to the p-values, regardless the value of p, the evidence can be very minimal or even supporting for the null-hypothesis [*27*]. These results demonstrate that without the computation of Bayes factors the interpretation of psychological results remains fairly limited.

It is to be noted, however, that our analyses cannot provide precise estimates to all psychological results, as they cover only the most frequently used statistical methods (t, and F statistics) and, furthermore, they are subject to the errors in the original reports and to the limitations of text-mining. In particular, it is important to highlight that the analyzed results certainly contain a proportion of manipulation check, therefore, the present results are not exclusive descriptors of focal results. An additional distortion of the sample can originate from the fact that several questionable research practices affect the likelihood of finding evidence for false hypotheses [*4*]. Ad hoc exclusion of outliers [*28*], selective reporting of p-values [*29*], and motivated stopping rules for data collection [*4*] have all been diagnosed in the field as additional factors that increase the rate of false discovery. The preference in publication culture for significant results over nonsignificant results [*3*] also increases the prevalence of false positive reports and incentivizes researchers to produce significant results. Although the exact impact of these biases is unknown, recent analyses revealed a high level of distortion in published statistical results [*5*, *30*].

Changes to statistical practices have been repeatedly proposed in recent decades [*31–34*]. Relying more on reported confidence intervals [*35*] or using lower decision thresholds for p-values (0.001 in *33*, 0.005) [*36*] have been recommended to improve the use of NHST statistics. Despite the many reform attempts, NHST is still misinterpreted and misused, at an increasing rate in certain areas [*37*]. While, certainly, the use of Bayes factors can only be part of the solution, their proponents emphasize that besides being able to quantify evidence against, and even for, the null-hypothesis, its use is also less prone to potential biases of multiple comparisons or optional stopping rules [*38*]. In fact, within this approach, for any study with an insufficient level of evidence, the researchers may continue to collect data until the results allow decisions to be made about the hypotheses [*39*]. Nevertheless, even without Bayesian calculations great improvement in existing practices can be achieved, for example by simply reporting the largest possible ‘post-experimental rejection ratio’ calculated from the p-value of any standard statistical test [*40*]. As the new statistical methods become easily applicable [*41*], researchers and journal editors may become convinced to extend the statistical toolbox of psychological research when evaluating the relationship between theories and their empirical evidence.

This study aimed to highlight that disregarding all the intentional or unintentional biases in research practice, a sizable proportion of published results labeled as “statistically significant” provide, in themselves, very little or no convincing evidence for the presence of the effect. From this aspect, it is not that surprising that previous ‘evidential’ findings fail to replicate, rather it is a necessary consequence of the threshold of acceptance having been set too low for the purported effects [*19*, 36]. As failed replications in recent years motivated psychological research to increase the transparency of research and openness of science, the present results should carry a further message to the field that changes to the customary research and analysis practices are inevitable.

## Supporting information

S1 Supplementary methods and results (DOC)

S1 Analysis main text (R code). (RMD)

S1 Analysis supporting information (R code). (RMD)

## References

[1] Open Science Collaboration. Estimating the reproducibility of psychological science. 2015;349(6251). 10.1126/science.aac4716 26315443

[2] Wagenmakers EJ, Beek T, Dijkhoff L, Gronau QF, Acosta A, Adams RB Jr, et al Registered Replication Report: Strack, Martin, & Stepper (1988). Perspectives on Psychological Science. 2016;11(6):917–28. doi: 10.1177/1745691616674458 27784749 10.1177/1745691616674458

[3] Franco A, Malhotra N, Simonovits G. Publication bias in the social sciences: Unlocking the file drawer. Science. 2014;345(6203):1502–5. doi: 10.1126/science.1255484 25170047 10.1126/science.1255484

[4] John LK, Loewenstein G, Prelec D. Measuring the prevalence of questionable research practices with incentives for truth telling. Psychol Sci. 2012;0956797611430953. 10.1177/0956797611430953 22508865

[5] Nuijten MB, Hartgerink CH, Assen MA, Epskamp S, Wicherts JM. The prevalence of statistical reporting errors in psychology (1985–2013). Behav Res Methods. 2015;1–22. 26497820 10.3758/s13428-015-0664-2 PMC5101263

[6] Neyman J, Pearson ES. On the problem of the most efficient tests of statistical hypotheses. Philos. Philos Trans R Soc A. 1933;231(694–706):289–337.

[7] Gigerenzer G. Mindless statistics. J Socio-Econ. 2004;33(5):587–606.

[8] Goodman S. A dirty dozen: twelve p-value misconceptions. Semin Hematol. 2008;45(3):135–40. doi: 10.1053/j.seminhematol.2008.04.003 18582619 10.1053/j.seminhematol.2008.04.003

[9] Colquhoun D. An investigation of the false discovery rate and the misinterpretation of p-values. Open Sci. 2014;1(3):140216. 10.1098/rsos.140216 PMC4448847 26064558

[10] Rouder JN, Morey RD, Speckman PL, Province JM. Default Bayes factors for ANOVA designs. J Math Psychol. 2012;56(5):356–74.

[11] Morey RD, Rouder JN. Bayes factor approaches for testing interval null hypotheses. Psychol Methods. 2011;16(4):406–19. doi: 10.1037/a0024377 21787084 10.1037/a0024377

[12] Verhagen J, Wagenmakers E-J. Bayesian tests to quantify the result of a replication attempt. J Exp Psychol Gen. 2014;143(4):1457–75. doi: 10.1037/a0036731 24867486 10.1037/a0036731

[13] Popper K. The logic of scientific discovery. Routledge, 2005.

[14] Rouder JN, Richard M, Wagenmakers E-J. The interplay between subjectivity, statistical practice, and psychological science. Collabra: Psychology 2016; 2, 1.

[15] Rouder JN, Speckman PL, Sun D, Morey RD, Iverson G. Bayesian t tests for accepting and rejecting the null hypothesis. Psychon Bull Rev. 2009;16(2):225–37. doi: 10.3758/PBR.16.2.225 19293088 10.3758/PBR.16.2.225

[16] Jeffreys H. Theory of probability Oxford, UK: Oxford University Press; 1961.

[17] Lee MD, Wagenmakers EJ. Bayesian cognitive modeling: A practical course. Cambridge University Press; 2014 4 3.

[18] Schönbrodt FD, Wagenmakers EJ, Zehetleitner M, Perugini M. Sequential hypothesis testing with Bayes factors: Efficiently testing mean differences. Psychological Methods. 2017;22(2):322 doi: 10.1037/met0000061 26651986 10.1037/met0000061

[19] Etz A, Vandekerckhove J. A Bayesian perspective on the reproducibility project: Psychology. PLoS ONE. 2016;11(2):e0149794 doi: 10.1371/journal.pone.0149794 26919473 10.1371/journal.pone.0149794 PMC4769355

[20] Vovk VG. A logic of probability, with application to the foundations of statistics. J R Stat Soc Ser B Methodol. 1993;317–51.

[21] Wetzels R, Matzke D, Lee MD, Rouder JN, Iverson GJ, Wagenmakers E-J. Statistical evidence in experimental psychology an empirical comparison using 855 t tests. Perspect Psychol Sci. 2011;6(3):291–8. doi: 10.1177/1745691611406923 26168519 10.1177/1745691611406923

[22] Hartgerink CH. 688,112 Statistical Results: Content Mining Psychology Articles for Statistical Test Results. Data. 2016;1(3):14.

[23] Morey RD, Rouder JN, Jamil T. BayesFactor: Computation of Bayes factors for common designs. R Package Version 09. 2014;8.

[24] Dienes Z. Bayesian versus orthodox statistics: Which side are you on? Perspect Psychol Sci. 2011;6(3):274–90. doi: 10.1177/1745691611406920 26168518 10.1177/1745691611406920

[25] Szucs D, Ioannidis JP. Empirical assessment of published effect sizes and power in the recent cognitive neuroscience and psychology literature. bioRxiv. 2016;071530. 10.1371/journal.pbio.2000797 PMC5333800 28253258

[26] Sellke T, Bayarri MJ, Berger JO. Calibration of ρ values for testing precise null hypotheses. Am Stat. 2001;55(1):62–71.

[27] Dienes Z, Mclatchie N. Four reasons to prefer Bayesian analyses over significance testing. Psychonomic Bulletin & Review. 2017 Mar 28:1–2. 10.3758/s13423-017-1266-z PMC5862925 28353065

[28] Simmons JP, Nelson LD, Simonsohn U. False-Positive Psychology Undisclosed Flexibility in Data Collection and Analysis Allows Presenting Anything as Significant. Psychol Sci. 2011 10 1;0956797611417632. 10.1177/0956797611417632 22006061

[29] Simonsohn U, Nelson LD, Simmons JP. P-curve: a key to the file-drawer. J Exp Psychol Gen. 2014;143(2):534–47. doi: 10.1037/a0033242 23855496 10.1037/a0033242

[30] Hartgerink CH, van Aert RC, Nuijten MB, Wicherts JM, van Assen MA. Distributions of p-values smaller than. 05 in psychology: what is going on? PeerJ. 2016;4:e1935 doi: 10.7717/peerj.1935 27077017 10.7717/peerj.1935 PMC4830257

[31] Johnson VE, Payne RD, Wang T, Asher A, Mandal S. On the reproducibility of psychological science. J Am Stat Assoc. 2016; 10.1080/01621459.2016.1240079 PMC5976261 29861517

[32] Pashler H, Wagenmakers E-J. Editors’ Introduction to the Special Section on Replicability in Psychological Science A Crisis of Confidence? Perspect Psychol Sci. 2012 11 1;7(6):528–30. doi: 10.1177/1745691612465253 26168108 10.1177/1745691612465253

[33] Berger JO, Sellke T. Testing a point null hypothesis: the irreconcilability of P values and evidence. J Am Stat Assoc. 1987;82(397):112–22.

[34] Ioannidis JP. Why most published research findings are false. PLoS Med. 2005;2(8):e124 doi: 10.1371/journal.pmed.0020124 16060722 10.1371/journal.pmed.0020124 PMC1182327

[35] Cumming G. The new statistics why and how. Psychol Sci. 2013;0956797613504966. 10.1177/0956797613504966 24220629

[36] Johnson VE. Revised standards for statistical evidence. Proc Natl Acad Sci. 2013;110(48):19313–7. doi: 10.1073/pnas.1313476110 24218581 10.1073/pnas.1313476110 PMC3845140

[37] Pritschet L, Powell D, Horne Z. Marginally Significant Effects as Evidence for Hypotheses Changing Attitudes Over Four Decades. Psychol Sci. 2016;0956797616645672. 10.1177/0956797616645672 27207874

[38] Dienes Z. How Bayes factors change scientific practice. J Math Psychol. 2016;72:78–89.

[39] Rouder JN. Optional stopping: No problem for Bayesians. Psychon Bull Rev. 2014;21(2):301–8. doi: 10.3758/s13423-014-0595-4 24659049 10.3758/s13423-014-0595-4

[40] Bayarri MJ, Benjamin DJ, Berger JO, Sellke TM. Rejection odds and rejection ratios: A proposal for statistical practice in testing hypotheses. J Math Psychol. 2016;72:90–103. 10.1016/j.jmp.2015.12.007 PMC6358203 30713353

[41] JASP Team. JASP (Version 0.8.0.0). 2016.

