# Supplementary material for: Estimating the evidential value of significant results in psychological science
Source: PLoS One. 2017 Aug 18;12(8):e0182651. doi: 10.1371/journal.pone.0182651 (PMC5562314; doi:10.1371/journal.pone.0182651)
Supplement: S1 Supplementary methods and results — (DOC) [file pone.0182651.s001.doc]

Supplementary Methods and Results

Dataset

For the analyses, we used a dataset collected by Hartgerink [*22*]. This dataset contains the metadata and statistical parameters of 688,112 null-hypothesis test results text-mined from 50,845 articles published in 321 psychology journals between 1985 and 2016 by American Psychological Association, Springer, Sage, and Taylor & Francis publishers. In addition to the extracted parameters, the dataset includes p-values recalculated from the reported test statistics and it is indicated whether a one-tailed test would produce the reported p-values. The dataset and the detailed description of the text-mining procedure are available on the following link: <http://dx.doi.org/10.17026/dans-zsk-k335>.

Preregistration

The analysis plan of this study was preregistered on osf.io/5rf2x prior the conduct of all the reported analyses. Two steps of the final analysis were not described in the pre-registration. First, we did not explicate the necessity of the below reported additional robustness checks. Second, we have not specified whether we would assume independent paired sample design for the calculation of the Bayes factors in our main analysis. This decision was made later, after considering that this way of computation is more lenient towards the H1 hypothesis.

Aims of the supplementary analyses

The aims of the supplementary analyses were twofold. First, we intended to ascertain the robustness of our results independently of the assumption of the design (within- vs. between-subjects) and the size of the prior scale of the Bayes factor. Second, we aimed to explore the extent to which the results of the main analyses could be generalized by replicating the analyses on a different test statistics, namely on correlational tests.

Data preparation for the supplementary analyses

In the supplementary analyses we employed the parameters of the t, F and r statistics from the dataset of Hartgerink [*22*] and conducted the same steps of data exclusion and transformation as for the main analyses. First, we selected the t-test statistics (169,984 t-tests), the F statistics (233,945 F-tests where the value of the first degrees of freedom was one) and the r statistics (21,820 r-tests). Next, we limited our database to the significant test statistics (exclusion of 115,534 results). We omitted the results lacking the exact values of the test statistics (380 results), as well as the one-sided tests (5488 results). Moreover, we removed those test statistics where the recalculated p-values changed whether or not the results were statistically significant using the 0.05 decision threshold (781 results). Finally, we excluded the r-tests with perfect correlations (r = 1; 7 results).

The steps of data transformation were the following: we rounded up all of the non-integer degrees of freedoms of the t-tests, we calculated the square root of the F-values to obtain the corresponding t values, we computed the square of the r values and we calculated the sample sizes of the tests from the reported degrees of freedoms. As the sample size calculation was based on the degrees of freedoms, we obtained different values when we assumed that the design of the t- and F-tests was independent-sampled compared to paired-sample. For the correlational test, we computed the sample size assuming only paired-samples design.

As a last step, we took out the results where the overall sample size did not exceed 5 to exclude the extreme cases. Assuming independent-samples design, we had to exclude 1,078 t- and F-tests, and so we retained 114,272 t-tests and 173,152 F-tests. Employing the assumption that the designs were paired-samples, we excluded 2,012 t- and F-tests, and 31 r-tests, leaving 113,773 t-tests, 172,717 F-tests and 15,026 r-tests in the dataset.

Supplementary Analyses

In order to gain the corresponding Bayes factors of the t- and F-tests, we applied the *ttest.tstat* function of the BayesFactor R package [*23*], and for the correlational analyses, we employed the *linearReg.R2stat* function of the same R package. As we had no information on the design of the obtained t- and F-tests, we calculated the Bayes factors assuming independent as well as paired-samples design. To explore the robustness of our results to the size and shape of the chosen prior distribution, we repeated all analyses with different priors. For the main analysis, we used the default settings of the employed functions to model the predictions of the alternative hypotheses (see the main text for the indication of this choice). The default prior of the t- and F-tests is a Cauchy distribution on the possible effect sizes, which is centered on zero and has a ‘medium’ scale (√2/2). The default prior of the regression analysis is a normal distribution on the regression slopes, and it is fitted on zero with a ‘medium’ standard deviation (√2/4). As the scale of the default priors is the lowest from the possible options in the package, we repeated the analyses with the relatively high “wide” scaled prior distributions (1 for t-tests and 1/2 for regressions). However, choosing a heavy-tailed prior distribution, such as the Cauchy, could overestimate the size of the possible effects, and so it could underrate the amount of evidence supporting the alternative hypotheses. Therefore, we reran the analyses of the t- and F-tests with a normally distributed prior. We fitted the distribution on zero and chose 0.5 as the standard deviation. For normally distributed priors, the double of the standard deviation represents the maximum effect size that we can expect to find. We based our decision about the size of the standard deviation of the prior on the work of Simmons and his colleagues [*42*], who estimated that high effect sizes usually vary around d = 1 and so we decided to use it as the maximum size of the potential effects.

**Results**

Table S1 presents the proportion of test results belonging to the different evidence categories for each type of calculation.

Finally, we calculated the strength of evidence for 10 equal ranges of the significant p-values of the t- and F-test results using `medium` scaled prior distributions and independent samples design, the most lenient conditions for supporting the alternative hypothesis. Table S2 summarizes the proportion of t- and F-test results broken down by the different p-value ranges and Bayes factor evidence categories.

These analyses indicate that the pattern of results is very similar using different statistics and the design or prior scale settings.

R script of supplementary analyses

The markdown R code of the supplementary analyses can be found at the following link: [https://www.dropbox.com/s/a5h0zo7v73icyws/Bayes%20Analysis%20Supplement.Rmd?dl=0](https://www.dropbox.com/s/a5h0zo7v73icyws/Bayes Analysis Supplement.Rmd?dl=0) [OSF link will be supplied after final acceptance]

**Table S1.**

The Proportion of test results in Bayes factor evidence categories calculated with both medium and wide scale prior across t-, F- and r- statistics.

|  |  |  | |  | **Bayes factor evidence categories** | | | | | |  |
| --- | --- | --- | --- | --- | --- | --- | --- | --- | --- | --- | --- |
|  |  | Prior distributions used | Type of data | | Strong H1 | Moderate H1 | Anecdotal H1 | Anecdotal H0 | Moderate H0 | Strong H0 | Total (N) |
| t and F tests | as independent samples design | Normal_SD=0.5_ | N/row total (%) | | **51.5%** | **28.9%** | **19.4%** | **0.2%** | **0.0%** | **0.1%** | 287,397[[1]](#footnote-2) |
|  |  |  | N | | 147,943 | 83,012 | 55,787 | 445 | 26 | 184 |  |
|  |  | Cauchy_medium_ | N/row total (%) | | **54.7%** | **21.4%** | **22.9%** | **1.0%** | **0.0%** | **0.0%** | 287,424 |
|  |  |  | N | | 157,184 | 61,579 | 65,677 | 2,861 | 116 | 7 |  |
|  |  | Cauchy_wide_ | N/row total (%) | | **54.4%** | **19.7%** | **23.2%** | **2.6%** | **0.1%** | **0.0%** | 287,424 |
|  |  |  | N | | 156,288 | 56,659 | 66,792 | 7,405 | 272 | 8 |  |
|  | as paired samples design | Normal_SD=0.5_ | N/row total (%) | | **56.9%** | **24.0%** | **17.9%** | **1.1%** | **0.0%** | **0.0%** | 286,466 |
|  |  |  | N | | 162,905 | 68,806 | 51,385 | 3,127 | 150 | 93 |  |
|  |  | Cauchy_medium_ | N/row total (%) | | **53.3%** | **17.8%** | **22.9%** | **5.7%** | **0.2%** | **0.0%** | 286,466 |
|  |  |  | N | | 152,823 | 51,037 | 65,611 | 16,359 | 637 | 23 |  |
|  |  | Cauchy_wide_ | N/row total (%) | | **51.6%** | **16.0%** | **21.6%** | **10.2%** | **0.6%** | **0.0%** | 286,466 |
|  |  |  | N | | 147,790 | 45,834 | 61,880 | 29,161 | 1,751 | 74 |  |
| r tests |  | Normal_medium_ | N/row total (%) | | **59.6%** | **19.6%** | **20.3%** | **0.5%** | **0.0%** | **0.0%** | 15,026 |
|  |  |  | N | | 8,950 | 2,945 | 3,052 | 79 | 0 | 0 |  |
|  |  | Normal_wide_ | N/row total (%) | | **59.1%** | **17.8%** | **21.6%** | **1.6%** | **0.0%** | **0.0%** | 15,026 |
|  |  |  | N | | 8,873 | 2,675 | 3,242 | 235 | 1 | 0 |  |

**Table S2.**

The proportion of t- and F-test results across p-value ranges and Bayes factor evidence categories. The Bayes factors were calculated with medium scaled prior distribution assuming independent-samples.

|  |  | **p-values** | | | | | | | | | |  |
| --- | --- | --- | --- | --- | --- | --- | --- | --- | --- | --- | --- | --- |
|  |  | **0.000-0.005** | **0.005-0.010** | **0.010-0.015** | **0.015-0.020** | **0.020-0.025** | **0.025-0.030** | **0.030-0.035** | **0.035-0.040** | **0.040-0.045** | **0.045-0.050** | **Row Total  n/ Table Total,** (n) |
| Strong H1 | N | 157,184 | 0 | 0 | 0 | 0 | 0 | 0 | 0 | 0 | 0 | **54.7%** (157,184) |
|  | N / Row Total | 100.0% | 0.0% | 0.0% | 0.0% | 0.0% | 0.0% | 0.0% | 0.0% | 0.0% | 0.0% |  |
|  | N / Col Total | 94.1% | 0.0% | 0.0% | 0.0% | 0.0% | 0.0% | 0.0% | 0.0% | 0.0% | 0.0% |  |
|  | N / Table Total | 54.7% | 0.0% | 0.0% | 0.0% | 0.0% | 0.0% | 0.0% | 0.0% | 0.0% | 0.0% |  |
| Moderate H1 | N | 9,683 | 25,142 | 16,192 | 8,904 | 1,656 | 0 | 0 | 2 | 0 | 0 | **21.4%** (61,579) |
|  | N / Row Total | 15.7% | 40.8% | 26.3% | 14.5% | 2.7% | 0.0% | 0.0% | 0.0% | 0.0% | 0.0% |  |
|  | N / Col Total | 5.8% | 97.5% | 88.7% | 60.5% | 13.0% | 0.0% | 0.0% | 0.0% | 0.0% | 0.0% |  |
|  | N / Table Total | 3.4% | 8.7% | 5.6% | 3.1% | 0.6% | 0.0% | 0.0% | 0.0% | 0.0% | 0.0% |  |
| Anecdotal H1 | N | 88 | 626 | 2,010 | 5,725 | 10,931 | 10,932 | 10,037 | 9,270 | 8,295 | 7,763 | **22.9%** (65,677) |
|  | N / Row Total | 0.1% | 1.0% | 3.1% | 8.7% | 16.6% | 16.6% | 15.3% | 14.1% | 12.6% | 11.8% |  |
|  | N / Col Total | 0.1% | 2.4% | 11.0% | 38.9% | 85.7% | 97.8% | 96.7% | 94.9% | 92.5% | 89.7% |  |
|  | N / Table Total | 0.0% | 0.2% | 0.7% | 2.0% | 3.8% | 3.8% | 3.5% | 3.2% | 2.9% | 2.7% |  |
| Anecdotal H0 | N | 2 | 27 | 55 | 77 | 155 | 233 | 319 | 485 | 644 | 864 | **1.0%** (2,861) |
|  | N / Row Total | 0.1% | 0.9% | 1.9% | 2.7% | 5.4% | 8.1% | 11.1% | 17.0% | 22.5% | 30.2% |  |
|  | N / Col Total | 0.0% | 0.1% | 0.3% | 0.5% | 1.2% | 2.1% | 3.1% | 5.0% | 7.2% | 10.0% |  |
|  | N / Table Total | 0.0% | 0.0% | 0.0% | 0.0% | 0.1% | 0.1% | 0.1% | 0.2% | 0.2% | 0.3% |  |
| Moderate H0 | N | 0 | 0 | 4 | 2 | 10 | 14 | 18 | 13 | 28 | 27 | **0.0%** (116) |
|  | N / Row Total | 0.0% | 0.0% | 3.4% | 1.7% | 8.6% | 12.1% | 15.5% | 11.2% | 24.1% | 23.3% |  |
|  | N / Col Total | 0.0% | 0.0% | 0.0% | 0.0% | 0.1% | 0.1% | 0.2% | 0.1% | 0.3% | 0.3% |  |
|  | N / Table Total | 0.0% | 0.0% | 0.0% | 0.0% | 0.0% | 0.0% | 0.0% | 0.0% | 0.0% | 0.0% |  |
| Strong H0 | N | 0 | 0 | 1 | 0 | 0 | 0 | 4 | 0 | 1 | 1 | **0.0%** (7) |
|  | N / Row Total | 0.0% | 0.0% | 14.3% | 0.0% | 0.0% | 0.0% | 57.1% | 0.0% | 14.3% | 14.3% |  |
|  | N / Col Total | 0.0% | 0.0% | 0.0% | 0.0% | 0.0% | 0.0% | 0.0% | 0.0% | 0.0% | 0.0% |  |
|  | N / Table Total | 0.0% | 0.0% | 0.0% | 0.0% | 0.0% | 0.0% | 0.0% | 0.0% | 0.0% | 0.0% |  |
| **Column Total** | **N / Table Total** | **58.1% 166,957** | **9.0% 25,795** | **6.4% 18,262** | **5.1% 14,708** | **4.4% 12,752** | **3.9% 11,179** | **3.6% 10,378** | **3.4% 9,770** | **3.1% 8,968** | **3.0% 8,655** | **287,424 (100%)** |
|  | **N** |  |  |  |  |  |  |  |  |  |  |  |

1. 27 cases have been deleted from this dataset, as the employed function failed to calculate the corresponding B values. [↑](#footnote-ref-2)
